# A Crosstalk between the Smad and JNK Signaling in the TGF-β-Induced Epithelial-Mesenchymal Transition in Rat Peritoneal Mesothelial Cells

**DOI:** 10.1371/journal.pone.0032009

**Published:** 2012-02-27

**Authors:** Qinghua Liu, Yu Zhang, Haiping Mao, Wei Chen, Ning Luo, Qin Zhou, Wenfang Chen, Xueqing Yu

**Affiliations:** Department of Nephrology, The First Affiliated Hospital, Sun Yat-sen University, Key Laboratory of Nephrology, Ministry of Health, Guangzhou, China; Massachusetts Eye & Ear Infirmary - Harvard Medical School, United States of America

## Abstract

Transforming growth factor β (TGF-β) induces the process of epithelial-mesenchymal transition (EMT) through the Smad and JNK signaling. However, it is unclear how these pathways interact in the TGF-β1-induced EMT in rat peritoneal mesothelial cells (RPMCs). Here, we show that inhibition of JNK activation by introducing the dominant-negative JNK1 gene attenuates the TGF-β1-down-regulated E-cadherin expression, and TGF-β1-up-regulated α-SMA, Collagen I, and PAI-1 expression, leading to the inhibition of EMT in primarily cultured RPMCs. Furthermore, TGF-β1 induces a bimodal JNK activation with peaks at 10 minutes and 12 hours post treatment in RPMCs. In addition, the inhibition of Smad3 activation by introducing a Smad3 mutant mitigates the TGF-β1-induced second wave, but not the first wave, of JNK1 activation in RPMCs. Moreover, the inhibition of JNK1 activation prevents the TGF-β1-induced Smad3 activation and nuclear translocation, and inhibition of the TGF-β1-induced second wave of JNK activation greatly reduced TGF-β1-induced EMT in RPMCs. These data indicate a crosstalk between the JNK1 and Samd3 pathways during the TGF-β1-induced EMT and fibrotic process in RPMCs. Therefore, our findings may provide new insights into understanding the regulation of the TGF-β1-related JNK and Smad signaling in the development of fibrosis.

## Introduction

Peritoneal fibrosis, a major complication of peritoneal dialysis, largely limits the effectiveness of peritoneal dialysis as a long-term renal replacement therapy [1]. Peritoneal fibrosis is attributed to the epithelial-mesenchymal transition (EMT) of peritoneal mesothelial cells [2], leading to the formation of submesothelial myofibroblasts associated with the accumulation of extracellular matrix (ECM) [3]. During the process of EMT, epithelial cells usually lose their adhesion ability because of the down-regulation of E-cadherin and β-catenin expression and the cytoskeletal rearrangement, resulting in up-regulated α-SMA expression and actin reorganization. This process further promotes the disruption of the basement membrane, and enhances cell migration and invasion [4], [5]. Increasing evidence indicates that transforming growth factor-β (TGF-β) can initiate the EMT process in peritoneal mesothelial cells, and is a key mediator of the dialysis-related peritoneal fibrosis [6], [7]. However, the mechanisms that regulate the TGF-β-induced EMT in peritoneal mesothelial cells are not fully understood.

The TGF-β/Smad signaling is critical for regulating the cell-state specific modulation. TGF-β binds to the type II receptor, which recruits and activates the type I receptor, leading to the downstream Smad2/3 phosphorylation and nuclear translocation and regulating the expression of target genes [8]. Previous studies have shown that the JNK/MAPK pathway regulates the TGF-β1-induced and Smad2-dependent IL-6 production in human bronchial epithelial cells [9] and modulates the TGF-β-induced transcriptional responses [10]. Many studies have suggested that the JNK signal pathway contributes to the fibrotic process in different models of diseases [11], [12]. Apparently, there is a mutual interaction between the TGF-β-related JNK and Smad pathways [13]–[16]. Our previous studies have demonstrated that the aberrant activation of the TGF-β/Smad signaling is associated with the peritoneal dialysis fluid-increased fibrotic peritoneum thickness, α-smooth muscle actin (α-SMA), collagen I, and plasminogen activator inhibitor-1 (PAI-1) expression, leading to the development of peritoneal fibrosis in rats [17], [18]. Our recent study has demonstrated that TGF-β1 induces the JNK and Smad3 activation and EMT in rat peritoneal mesothelial cells (RPMCs) [19]. However, the role of the JNK1 signal pathway in the TGF-β1-induced EMT and the interaction between the JNK1 and Smad3 pathways in RPMCs remain poorly understood.

In the present study, we examined the impact of inducing dominant-negative JNK1 expression on the TGF-β1–induced Smad3 activation and EMT in RPMCs, and determined the effect of inducing mutant Smad3 expression on the TGF-β1-induced JNK activation in RPMCs.

## Results

### JNK1 mediates the TGF-β1-induced EMT of RPMCs

**Figure 1:**
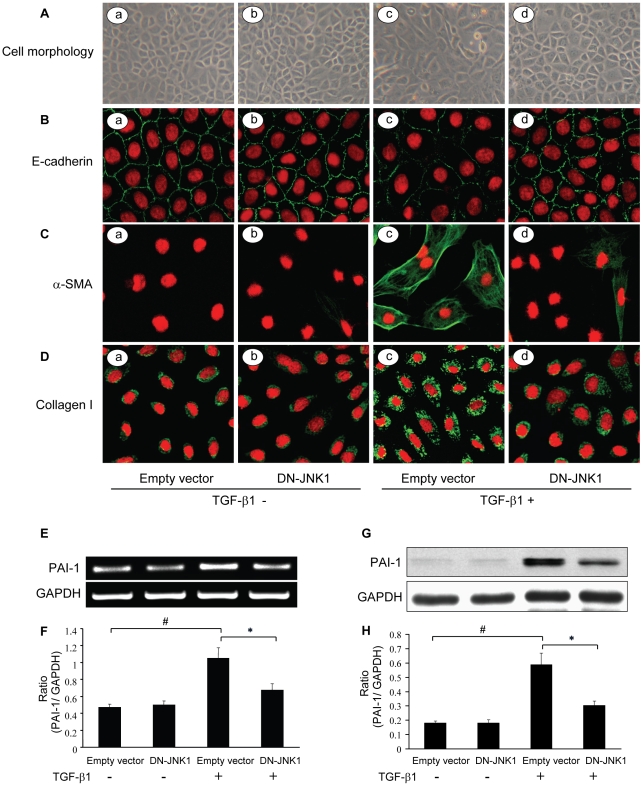
The JNK1 regulates the TGF-β1-induced EMT process in RPMCs. RPMCs were infected with Ad-DN-JNK1 or Ad-control for 24 h, and stimulated with, or without, 10 ng/ml of TGF-β1 for 24 or 48 hours, respectively. Their morphologies, E-cadherin, α-SMA, Collagen I, and PAI-1 expression were determined by histology, immunofluorescence, RT-PCR, and Western blot assays, respectively. (A) Photomicrographs of confluent monolayer of RPMCs (magnification ×200). (B–D) Immunofluorescence analyses of E-cadherin (B), α-SMA (C) and Collagen I (D). After stimulating with TGF-β1 for 48 hours, the cells were fixed with 4% paraformaldehyde, and stained with the specific FITC-conjugated antibodies, followed by counterstaining of the nucleus with DAPI (magnification ×400). (E) RT-PCR analysis of the PAI-1 and GAPDH mRNA transcripts. (F) Quantitative analysis of the relative levels of PAI-1 mRNA transcripts to control GAPDH by densitometric scanning. (G) Western blot analysis of the PAI-1 protein. The cell lysates from different groups of cells that had been stimulated with TGF-β1 for 48 hours were subjected to Western blot analysis using the specific antibodies against PAI-1 and GAPDH. (H) Quantitative analysis of the relative levels of PAI-1 protein to control GAPDH by densitometric scanning. Data are expressed as representative images or as mean ± SEM of each group of cells from at least three separate experiments. * *P*<0.05, ^#^ *P*<0.01.

To analyze whether JNK1 could modulate the TGF-β1-induced EMT, RPMCs were infected with Ad-DN-JNK1 (Recombinant adenovirus vectors that expressed a dominant-negative JNK1) or Ad-control (control recombinant adenovirus), and treated with, or without, TGF-β1 for 48 hours. The cell morphology and the EMT-related myofibroblastic conversion were characterized by histology and immunofluorescency. As shown in Figure 1A, infection with Ad-DN-JNK1 alone did not modulate RPMC morphology, as epithelial cell morphology with a cobblestone-like growth pattern was observed in the Ad-control and Ad-DN-JNK1-infected cells in the absence of TGF-β1 treatment (Figure 1A, a and b). Following treatment with TGF-β1, many mesothelial cells that had been infected with Ad-control became spindle fibroblast-like cells (Figure 1A, c), indicative of myofibroblastic conversion. In contrast, RPMCs that had been infected with Ad-DN-JNK1 remained epithelial cell morphology, indicating that JNK1 is crucial for the TGF-β1–induced myofibroblastic conversion in RPMCs (Figure 1A, d). Confocal immunofluorescence analysis revealed that treatment with TGF-β1 reduced E-cadherin expression and disrupted the cell-cell interactions (Figure 1B, c), as compared with unstimulated control cells (Figure 1B, a). Furthermore, although infection with Ad-DN-JNK1 did not affect E-cadherin expression in the absence of TGF-β1 (Figure 1B, b), infection with Ad-DN-JNK1 resulted in a resistance of RPMCs to the TGF-β1-induced redistribution of E-cadherin (Figure 1B, d). In addition, treatment of RPMCs with TGF-β1 induced a remarkable reorganization of the microfilament network and induced stress fiber formation, determined by anti-α-SMA staining (Figure 1C, c). In contrast, only little stress fiber formation was detected in the TGF-β1-treated and Ad-DN-JNK1-infected RPMCs (Figure 1C, d), but not in the TGF-β1-untreated cells (Figure 1C, a and b). Moreover, treatment with TGF-β1 enhanced Collagen I expression in RPMCs (Figure 1D, c), but not in the Ad-DN-JNK1-infected cells (Figure 1D, d), as compared with that in the control cells (Figure 1D, a and b).

Analysis of the relative levels of endogenous PAI-1 transcripts revealed that treatment with TGF-β1 for 24 hours significantly elevated the relative levels of PAI-1 mRNA transcripts, as compared with that in the TGF-β1-untreated cells (Figure 1E and F). However, the inducing effect of TGF-β1 on the PAI-1 transcription was mitigated by infection with Ad-DN-JNK1 in RPMCs. Similar patterns of PAI-1 protein expression were detected in different groups of cells (Figure 1G and H). Collectively, these data indicate that TGF-β1 stimulates EMT and that JNK1 activity is crucial for the TGF-β1-induced EMT in primary cultured RPMCs.

### TGF-β1 stimulates bimodal JNK activation with rapid, transient, and gradual sustained peaks of activity

**Figure 2:**
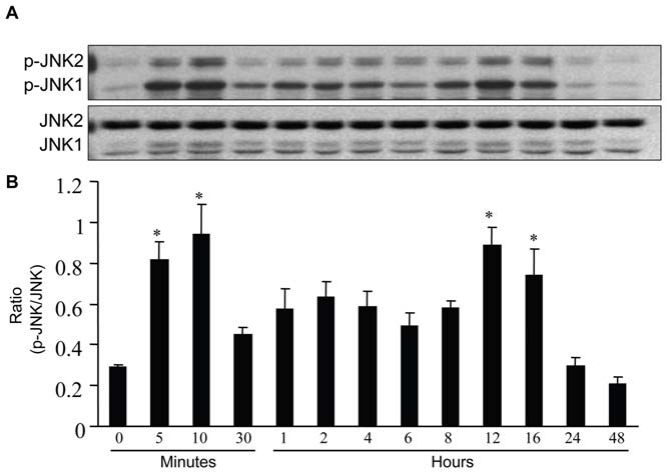
TGF-β1 stimulates a bimodal JNK activation in RPMCS. RPMCs were stimulated with 10 ng/ml of TGF-β1 in FBS-free DMEM/F12, and the cells were harvested at the indicated time points. The relative levels of JNK1, JNK2, phosphorylated JNK1 and JNK2 were determined by Western blot assays using the specific antibodies. (A) Western blot analysis of JNK1, JNK2, phosphorylated JNK1 and JNK2. (B) Quantitative analysis. The relative ratios of phosphorylated JNK to JNK were determined by densitometric scanning. Data are expressed as representative images or as mean ± SEM from at least three separate experiments. * *P*<0.05 vs. the value at 0 time point.

Our previous study has demonstrated that JNK can be activated by TGF-β1 and that the highest activity of JNK occurs at 10 minutes post treatment in RPMCs [19]. However, it has also been reported that TGF-β1 may induce bimodal JNK activation in mink lung epithelial cells [14]. To determine the kinetics of the TGF-β1-induced JNK activation, RPMCs were treated with TGF-β1 and the levels of JNK1 and JNK2 phosphorylation were characterized longitudinally by Western blot assays. As shown in Figure 2, although the levels of JNK1 and JNK2 expression were similar throughout the time course, the levels of phosphorylated JNK1 and JNK2 appeared to be higher at 5–10 minutes and 12–16 hours post TGF-β1 treatment. The relative ratios of phosphorylated JNK to JNK at 5–10 minutes and 12–16 hours post treatment were significantly greater than that of the other time points. Therefore, TGF-β1 induces two phases of JNK activation in RPMCs *in vitro*.

### TGF-β1-induced secondary peak of JNK1/2 activation, but not the first JNK1/2 activation, is mediated by Smad3 signaling

**Figure 3:**
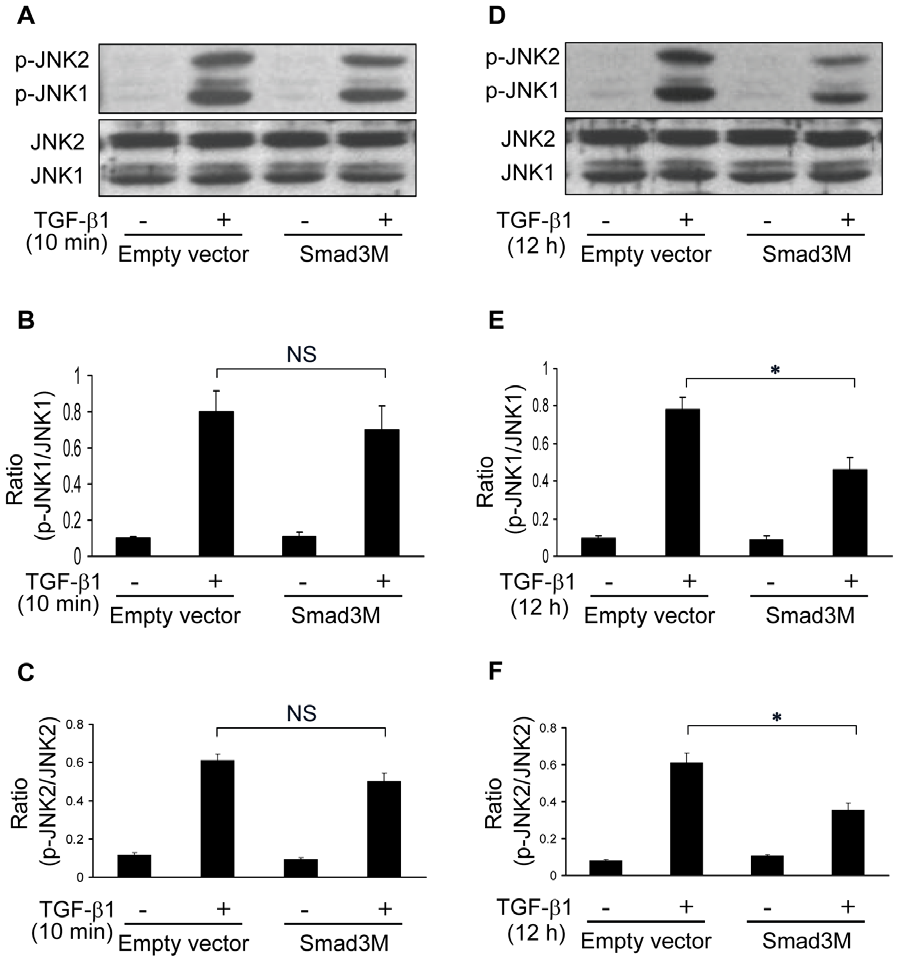
The Smad3 regulates the TGF-β1-induced second wave, but not the first wave, of JNK activation. RPMCs were transiently transfected with the pcDNA3-SamdM or empty vector pcDNA3 for 24 hours and treated with, or without, 10 ng/ml of TGF-β1 for 10 minutes or 12 hours, respectively. The relative levels of JNK1, JNK2, phosphorylated JNK1 and JNK2 were determined by Western blot assays. (A) Western blot analysis of the JNK activation at 10 minutes post TGF-β1 treatment. (B–C) Quantitative analysis. The relative levels of P-JNK1 to JNK1 (B) and p-JNK2 to JNK2 (C) were determined by densitometric scanning. (D) Western blot analysis of the JNK activation at 12 hours post TGF-β1 treatment. (E–F) Quantitative analysis. The relative levels of P-JNK1 to JNK1 (E) and p-JNK2 to JNK2 (F) were determined by densitometric scanning. Data are expressed as representative images or as mean ± SEM of at least three separate experiments. * *P*<0.05, NS: Not significant.

To test how the Smad3 affects the TGF-β1-induced JNK1/2 activation, RPMCs were transfected with vehicle pcDNA3 or pcDNA3-Smad3M carrying mutations at the C-terminus phosphorylation sites (ser422/425) of the Smad3, and then treated with, or without, TGF-β1 for 10 minutes or 12 hours, respectively. The relative levels of JNK1 and JNK 2 phosphorylation in those cells were determined by Western blot assays (Figure 3). There was no significant difference in the relative levels of phosphorylated JNK1 and JNK2 in the vehicle and pcDNA3-Smad3M-transfected cells at 10 minutes post TGF-β1 treatment (Figure 3A–C). In contrast, the relative levels of phosphorylated JNK1 and JNK2 in the pcDNA3-Smad3M-transfected cells were significantly lower than that of the controls at 12 hours post TGF-β1 treatment (Figure 3D–F). These data suggest that the Smad3 is important for TGF-β1 to induce the second peak of JNK activation, but not the first peak of JNK activation, in RPMCs in our experimental system.

### JNK1 regulates the TGF-β1-stimulated Smad3 phosphorylation and nuclear translocation

**Figure 4:**
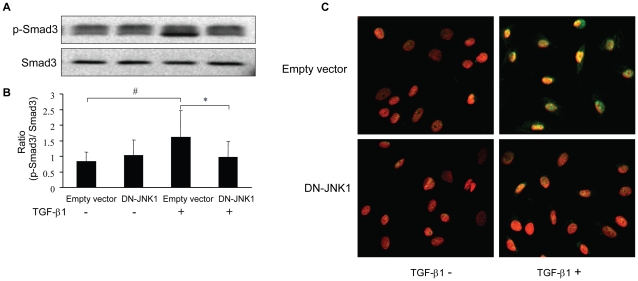
The JNK1 regulates the TGF-β1-induced Smad3 activation and nuclear translocation. RPMCs were infected with Ad-DN-JNK1 or Ad-control for 24 hours and treated with, or without, 10 ng/ml of TGF-β1 for 30 minutes. The relative levels of Smad3 and phosphorylated Smad3 and the nucleus p-Smad3 were characterized by Western blot and immunofluorescence using FITC-anti-p-Smad3 and DAPI for counterstaining of the nuclei. (A) Western blot analysis. (B) Quantitative analysis. The relative levels of phosphorylated Smad3 to Smad3 were determined by densitometric scanning. Data are expressed as representative images or as mean ± SEM of at least three separate experiments. * *P*<0.05, ^#^ *P*<0.01. (C) Immunofluorescence analyses of p-Smad3. The cells were fixed with 4% paraformaldehyde, and stained with FITC-anti-p-Smad3, followed by counterstained with DAPI for the nuclei. The monolayers were visualized and imaged with a laser scanning confocal microscope (p-Smad3: green, nucleus: red).

To dissect the role of JNK1 in the TGF-β1-induced Smad3 activation, RPMCs were infected with Ad-DN-JNK1 or Ad-control, and then treated with, or without, TGF-β1 for 30 minutes, respectively. The relative levels of phosphorylated Smad3 were determined by Western blot assays (Figure 4). Infection with Ad-control did not modulate the Smad3 expression and phosphorylation (Figure 4A and B). Treatment with TGF-β1 significantly elevated the relative levels of phosphorylated Smad3 in the vehicle-infected cells, but not in the Ad-DN-JNK1 infected cells. Furthermore, TGF-β1 effectively induced the p-Smad3 nuclear translocation in the Ad-control-infected cells, but not in the Ad-DN-JNK1-infected cells because strong staining of FITC-anti-p-Smad3 was observed predominately in the nuclei of Ad-control-infected RPMCs (Figure 4C). Together, these data suggest that JNK1 regulates the TGF-β1-induced Smad3 phosphorylation and nuclear translocation in RPMCs *in vitro*.

### Blockade of the second wave of TGF-β1-induced JNK activation mitigates the TGF-β1-induced EMT in RPMCs

**Figure 5:**
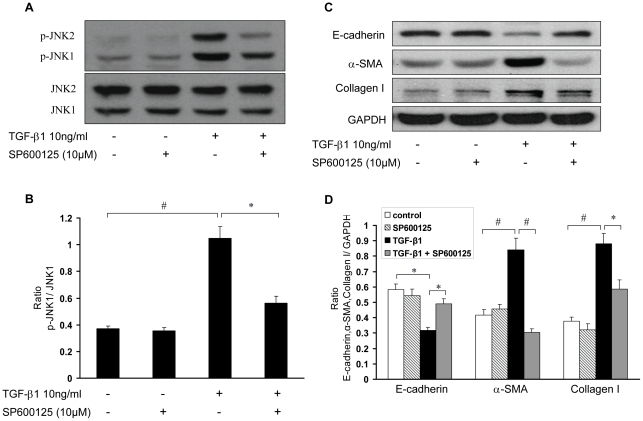
The second wave of JNK activation is crucial for the TGF-β1-induced EMT in RPMCs. RPMCs were stimulated with, or without, 10 ng/ml of TGF-β1 for 8 hours and treated with, or without, 10 µM SP600125 (a specific inhibitor of JNK). The cells were harvested at 12 hours (A, B) or 48 hours (C, D) post TGF-β1 stimulation. The relative levels of phosphorylated JNK1/2, and JNK1/2, E-cadherin, α-SMA, and Collagen I expression were determined by Western blot assays and densitometric scanning. (A) Western blot analysis of JNK1/2 and phosphorylated JNK1/2. (B) Quantitative analysis of the relative levels of phosphorylated JNK1/2 to JNK1/2. (C) Western blot analysis of E-cadherin, α-SMA, and Collagen I proteins. (D) Quantitative analysis of the relative levels of E-cadherin, α-SMA, and Collagen I proteins to control GAPDH. Data are expressed as representative images or as mean ± SEM of each group of cells from at least three separate experiments. * *P*<0.05, ^#^ *P*<0.01.

To determine whether the second wave of TGF-β1-induced JNK activation is involved in the TGF-β1-induced EMT in RPMCs, we stimulated RPMCs with TGF-β1 for eight hours and then treated with 10 µM SP600125, a specific JNK inhibitor, followed by characterizing the JNK phosphorylation at 12 hours and EMT in RPMCs at 48 hours post TGF-β1 treatment. We found that treatment with SP600125 effectively inhibited TGF-β1-induced JNK1/2 phosphorylation at 12 hours post TGF-β1 treatment (Figure 5 A and B) and significantly mitigated the TGF-β1-down-regulated E-cadherin expression and TGF-β1-enhanced α-SMA and Collagen I expression in RPMCs (Figure 5 C and D). Apparently, the second wave of JNK activation induced by TGF-β1 is important for TGF-β1-induced EMT in RPMCs.

## Discussion

In the present study, we examined the roles of the JNK1 pathway in the TGF-β1-induced EMT and explored the crosstalk between the JNK1 and Smad3 pathways in RPMCs. Our results indicated that JNK1 positively regulated the TGF-β1-down-regulated E-cadherin expression and up-regulated α-SMA, collagen I, and PAI-1 expression as well as myofibroblastic conversion in RPMCs. Furthermore, we found that TGF-β1 induced the JNK activation in a bimodal manner and that the TGF-β1-induced second peak, but not the first peak, of JNK activation appeared to be supported by Smad3 in RPMCs. Moreover, we found that JNK1 was critical for the TGF-β1-induced Smad3 phosphorylation and nuclear translocation in RPMCs. These data extended previous findings and suggested a mutual interaction between the TGF-β1-related JNK1 and Smad3 signal pathways during the EMT process in RPMCs.

Following the process of EMT, epithelial cells usually lose their adhesion ability because of the down-regulation of E-cadherin and β-catenin expression, cytoskeletal rearrangement, up-regulated α-SMA expression, and actin reorganization. The EMT of peritoneal mesothelial cells contributes to the development of peritoneal fibrosis [2], [20]. The EMT process is regulated by the TGF-β/Smad signaling and the MAPK/JNK signaling. Our previous study and those of others have demonstrated that inhibition of JNK activation effectively mitigates the TGF-β1–upregulated α-SMA, connective tissue growth factor (CTGF), and Collagen I expression, and down-regulated E-cadherin expression as well as the EMT process in different cell models [19], [21]–[23]. In the present study, we found that inhibition of JNK1 activity effectively attenuated the TGF-β1-down-regulated E-cadherin expression, but up-regulated α-SMA, Collagen I, and PAI-1 expression in RPMCs. Therefore, the JNK1 regulates the TGF-β1-induced EMT in primary cultures of RPMCs. Actually, the JNK1 has been shown to regulate the TGF-β1-stimulated fibronectin expression and EMT in human fibrosarcoma derived cells and mouse tracheal epithelial cells [24], [25], and the TGF-β1–induced expression of profibrotic genes *in vivo* [12]. Apparently, the JNK1 is a critical regulator of the process of EMT. We are interested in investigating the role of the JNK2 in the process of EMT during the development of peritoneal fibrosis.

PAI-1 is one of the major targets regulated by the TGF-β signal pathway [26] and has been thought to regulate the ECM turnover by preventing plasmin generation and plasmin-mediated MMP activation [27]. Up-regulated PAI-1 expression is associated with the development of peritoneal fibrosis [28], and the TGF-β1-stimulated PAI-1 expression is regulated by the JNK1 [24]. We found that inhibition of JNK1 mitigated the TGF-β1-stimulated PAI-1 expression in RPMCs, consistent with a previous observation that knockdown of JNK1 expression diminished the TGF-β1-induced PAI-1 expression in epithelial cells [25]. Notably, previous studies have shown that the JNK upstream and other MAPK signal events, such as MEKK-1, ERK, and p38MAPK, are crucial regulators of the TGF-β1-stimulated PAI-1 expression in different types of epithelial and mesangial cells [15], [29]–[31]. It is possible that blockage of MEKK1 activation minimizes the JNK activation, leading to inhibition of the TGF-β1-stimulated PAI-1 expression. Given that inhibition of JNK1 activation attenuated the Smad3 activation, the JNK1 may be a major regulator of the TGF-β1-stimulated PAI-1 expression in RPMCs.

The expression and deposition of ECM and Collagen I are important for the development of peritoneal fibrosis. The expression of Collagen I is positively regulated by the JNK activation. We found that inhibition of JNK1 activation mitigated the TGF-β1-stimulated Collagen I expression in RPMCs. Our data are consistent with previous findings that treatment with an inhibitor of the JNK activation prevented the TGF-β1-stimualted Collagen I expression [19], [32]. However, our data argued against the findings that the ERK pathway, but not the JNK pathway, is critical for regulating the TGF-β1-mediated collagen I expression in human mesangial cells [33] and that knockdown of JNK1 does not affect the TGF-β1-induced collagen I expression in pulmonary fibroblasts [34]. In addition, inhibition of the p38MAPK activation reduces the TGF-β1-mediated collagen I expression in hepatic stellate cells [35]. These disparate results suggest that different MAPK pathways regulate the TGF-β-stimulated ECM-related gene expression in different types of cells.

Many studies have shown that TGF-β1 can induce the JNK activation. However, the pattern of the TGF-β1-induced JNK activation remains in debate. While majority of studies indicates that TGF-β1 induces a rapid JNK activation, peaking at 10–30 minutes, and declining 1 hour post treatment [19], [22], [29], [36], [37], another study reveals that TGF-β1 induces a bimodal JNK activation in mink lung epithelial cells [14]. We determined the kinetics of TGF-β1-induced JNK activation and found that the first wave of JNK activation induced by TGF-β1 occurred as described previously [19]. However, TGF-β1 induced a second wave of JNK activation between 12–16 hours post treatment. Atfi A et al. [38] reported that TGF-β1 induced a delayed wave of JNK activation, with maximal activation at 12 hours, but not a rapid wave of JNK activation. On the contrary, another study suggests that TGF-β1 fails to induce the JNK activation *in vitro* [39]. The difference may stem from varying types of cells and diverse experimental conditions. More importantly, we found that inhibition of the second wave of JNK activation mitigated the TGF-β1-down-regulated E-cadherin, and enhanced α-SMA and Collagen I expression in RPMCs. These data further indicated the importance of JNK in regulating TGF-β1-induced EMT. These findings are supported by a recent report that knockdown of JNK expression inhibits the TGF-β1-induced CTGF and collagen I expression in telomerase-immortalized human cornea stroma fibroblast cells [40]. However, inhibition of the second wave of JNK activation failed to completely diminish TGF-β1-induced EMT in RPMCs, suggesting that other pathways may also contribute to the pathogenesis of EMT [41].

**Figure 6:**
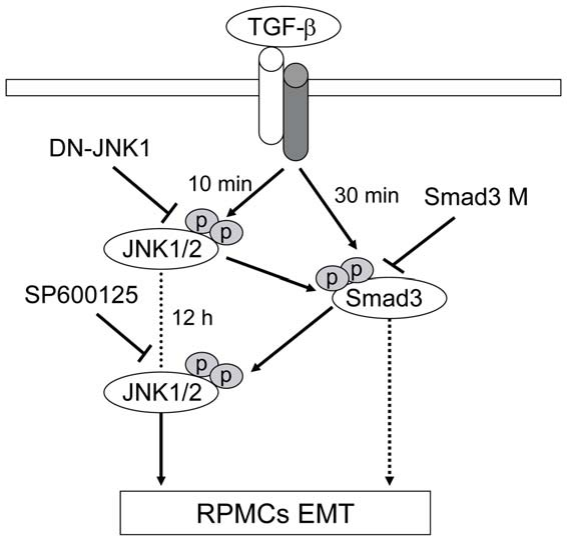
Schematic illustration of a crosstalk between Smad3 and JNK signaling in TGF-β-induced EMT in RPMCs. The diagram is generated, according to our findings from this study. RPMCs, Rat peritoneal mesothelial cells; DN-JNK1, Recombinant adenovirus vectors expressing dominant-negative JNK1 gene; Smad3M, pcDNA3-Smad3 carrying mutations at the C-terminus phosphorylation sites (ser422/425); SP600125, a specific inhibitor of JNK.

Our previous studies have demonstrated that the TGF-β/Smad pathway plays a crucial role in the development of peritoneal dialysis fluid-induced peritoneal fibrosis in rats [17], [18]. The Smad3 is a signal transducer, and the Smad3-deficient mice are resistant to the TGF-β1-induced peritoneal fibrosis [42]. Given that TGF-β1 activates both the Smad3 and JNK pathways, we examined the potential interaction between these pathways in the TGF-β1-induced EMT process in RPMCs. We found that inhibition of the Smad3 activation by introducing a mutant Smad3M significantly mitigated the TGF-β1-induced JNK activation at 12 hours, but not 10 minutes, post treatment, consistent with previous studies in other cells [14], [43]. These data reveal for the first time that, in primary cultures of RPMCs, Smad3 signaling reinforces the JNK pathway by augmenting the second wave of JNK activation induced by TGF-β1. The failure of Smad3 to regulate the TGF-β1-induced early JNK activation likely stems from the fact that TGF-β1 induces the Smad3 peak activation at 30 minutes post treatment [19]. Furthermore, we found that inhibition of the JNK1 activation dramatically reduced the TGF-β1-induced Smad3 activation and almost abolished Smad3 nuclear translocation in RPMCs. In addition, inhibition of the second wave of JNK1 activation induced by TGF-β1 dramatically mitigated the TGF-β1-induced EMT in RPMCs. Apparently, TGF-β1 induces Smad3 activation and the first wave of JNK1 activation. The activated JNK1 enhances the TGF-β1-related Smad3 activation and nuclear translocation, which in turn enhances the second wave of JNK activation, leading to a cascade of TGF-β1-related signaling and downstream EMT-related gene expression in RPMCs (Figure 6). The crosstalk between the TGF-β1-induced JNK and Samd3 pathways are supported by previous findings [34], [44], [45]. Notably, a previous study has shown that the JNK-dependent Jun activation inhibits TGF-β-Smad3 activation and downstream signaling [46] and that the JNK can act as a repressor of the TGF-β-mediated biological responses [47]. Indeed, we found that inhibition of the JNK1 activation only partially, but did not completely block the TGF-β1-induced Samd3 signaling in RPMCs. It is possible that other undescribed signal pathways regulate the TGF-β1-induced Samd3 signaling. Together, the present findings indicate a crosstalk between the Smad and JNK pathways during the TGF-β1-induced EMT and fibrotic process in RPMCs.

In summary, our data indicate that the JNK1 is a crucial amplifier of TGF-β1 signaling in promoting EMT in RPMCs. Furthermore, our data demonstrated a crosstalk between the JNK and Smad3 pathways during the TGF-β1-induced EMT and fibrotic process in RPMCs. Therefore, our findings may provide new insights into understanding the regulation of the TGF-β1-medaited signaling during the process of EMT and fibrosis in RPMCs.

## Methods

### Antibodies and Reagents

Human recombinant TGF-β1 was from R&D Systems (Minneapolis, MN, USA); antibodies against p-JNK (Thr183/Tyr185), JNK, p-Smad3 (Ser423/425)/Smad1 (Ser463/465), GAPDH, and control IgG were purchased from Cell Signaling (Beverly, MA, USA). Additional antibodies against Smad3 and E-cadherin were from Upstate (Lake Placid, NY, USA) and BD Biosciences (San Jose, CA, USA), respectively. Antibodies against PAI-1 and Type I Collagen were from Santa Cruz Biotechnology (Santa Cruz, CA, USA) and Southern Biotech (Birmingham, AL, USA), respectively. Monoclonal antibody against smooth muscle actin was from Sigma (St. Louis, Missouri, USA). Horseradish peroxidase (HRP)-conjugated goat anti-mouse IgG, donkey anti-rabbit IgG, Alexa Fluro 546-conjugated goat anti-rabbit IgG, Alexa Fluro 488-conjugated goat anti-mouse IgG, and Alexa Fluor 546-conjugated donkey anti-goat IgG were purchased from Cell Signaling. 4, 6-diamidino-2-phenylindole (DAPI) was purchased from Molecular Probes (Leiden, The Netherlands). SP600125 (a JNK inhibitor) was from Calbiochem (San Diego, CA, USA). Dulbecco's modified Eagle's medium (DMEM)/F12 and Fetal Bovine Serum (FBS) were purchased from Gibco-BRL (Grand Island, NY, USA). The RT-PCR kit and TRIZOL reagent were purchased from Invitrogen (San Diego, CA, USA).

### RPMCs isolation and culture

RPMCs were isolated and cultured as described previously [19]. Briefly, male Sprague-Dawley (SD) rats at 150–250 g were purchased from the Animal Experimental Center of Sun Yat-sen University, Guangzhou, Guangdong, China. Individual rats were injected intraperitoneally with 25–40 ml of 0.25% trypsinase-0.02% EDTA-Na_2_, and two hours later, the abdominal fluid of individual rats was collected, followed by centrifuging at 151× g for 10 minutes. The cells were cultured in 15% (v/v) FBS DMEM/F12 medium in 25 cm^2^ tissue culture flasks at 37°C in a humidified 5% CO_2_ atmosphere. The cells were passaged every 3–5 days, and RPMCs from the second and third passages at 80% confluence were used for the following experiments. The experimental protocols were approved by the Animal Care and Use Committee of the Sun Yat-Sen University. The approval ID for the animals used in this study is SCXK (Guangdong) 2006-0015.

### Plasmid constructs and transfection

Rat wild-type Smad3 plasmid (pcDNA3.0-Myc-Smad3) was generously provided by Prof. Huiyao Lan (University of Hongkong, Hong Kong, China). The Smad3M carrying mutations at the C-terminus phosphorylation sites (ser422/425) was generated by polymerase chain reaction–based mutagenesis using primers of forward 5′-TGGGTACCATGGAGCAAAAGCTAATAT-3′, and reverse 5′-GCACTCGAGCTAACAGCGGATGCTTGGGGA-3′, and subcloned into pcDNA3.0, followed by sequencing. PCR amplifications were performed at 95°C for 5 minutes and then 35 cycles of 94°C for 30 seconds, 53°C for 30 seconds, and 72°C for 45 seconds. RPMCs at 3×10^5^ cell/well were cultured overnight in 6-well plates, transfected in duplicate with 1 µg/well of pcDNA3.0-Smad3M or control pcDNA3.0 using Lipofectamine 2000 (Invitrogen) for 24 hours, and the cells were stimulated with TGF-β1 (10 ng/ml) for 10 minutes or 12 hours, respectively.

### Virus infection

Recombinant adenovirus vector (Ad-DN-JNK1) expressing dominant-negative JNK1 was a gift from Dr. Borkan (Boston University, Boston, Massachusetts, USA). RPMCs at 2×10^5^/well were cultured overnight in 6-well plates and infected in duplicate with 2×10^7^ PFU of Ad-DN-JNK1 or Ad-control for 24 hours, and were subjected to other treatments as indicated in the figure legends.

### RNA isolation and RT-PCR

Total RNA was extracted from RPMCs using the TRIZOL kit as described previously [19], and reversely transcribed into cDNA using the first-strand synthesis kit (Invitrogen). PCR amplifications of specific DNA fragments were performed in duplicate with TaqDNA polymerase and specific primers. The sequences of primers were designed based on published GenBank sequences and sense 5′-TACGACATCCTGGAACTGCC-3′, antisense 5′-GGAGGAAGACGCCACTGT-3′ for rat PAI-1; sense 5′-GGCAAGTTCAATGGCACAGT-3′, and antisense 5′-AAGGTGGAGGAATGGGAGTT-3′ for GAPDH. PCR amplifications were performed at 94°C for 5 minutes, and then 30 and 29 cycles of 94°C for 30 seconds, 56°C for 30 seconds, and 72°C for 45 seconds, followed by extension at 72°C for 10 minutes for PAI-1 and GAPDH, respectively. The PCR products were resolved electrophoretically on 1.2% agarose gels containing 0.05 µg/ml of ethidium bromide, and imaged using an Alpha Fluorchem TM 8900 auto-image system (Alpha Innotech, San Leandro, USA).

### Western blot analysis

Different groups of RPMCs were lyzed in lysis buffer (Cell Signaling), and after centrifuging, the cell lysates proteins were determined by the Bradford protein assay (Bio-Rad, Hercules, CA, USA). The cell lysates proteins (30–50 µg/lane) were separated by 12% SDS-PAGE and transferred to nitrocellulose membranes. After blocking with 5% fat-free dried milk in TBST, the membranes were incubated overnight at 4°C with anti-PAI-1, anti-p-JNK, anti-JNK, anti-p-Smad3, anti-Smad3, anti-E-cadherin, anti-α-SMA, anti-Collagen I, anti-GAPDH, or 5 µg/ml of control IgG, respectively. The bound antibodies were detected by appropriate HRP-conjugated IgG and visualized using an enhanced chemiluminescence system (Kodak, Rochester, NY, USA). The relative levels of each protein to control GAPDH were determined by densitometric analysis.

### Immunofluorescence study

RPMCs at 2×10^5^/well were cultured overnight on glass cover-slips in 3.5 cm-diameter tissue culture plates. The cells were starved in serum-free medium for 15 hours and infected with 2×10^7^ PFU of Ad-DN-JNK1 or Ad-control for 24 hours in 0.2% FBS DMEM/F12, respectively. The cells were treated in duplicate with 10 ng/ml of TGF-β1 for 30 minutes (to detect phospho-smad3) or 48 hours (to detect E-cadherin, α-SMA, and Collagen I), fixed with 4% paraformaldehyde, and permeabilized in 0.1% Triton X-100 for 10 minutes at room temperature, followed by blocking with 5% BSA in PBS. Subsequently, the cells were probed with polyclonal rabbit anti-phospho-smad3 (1∶50), monoclonal mouse anti-E-cadherin (1∶100), anti-α-SMA (1∶100), polyclonal goat anti-Collagen I (1∶100), or control IgG at 4°C overnight, respectively. After washing, the bound antibodies were detected with Alexa Fluro 488-conjugated anti-mouse IgG (1∶1000), Alexa Fluro 546-conjugated anti-rabbit IgG (1∶1000), or Alexa Fluor 546-conjugated anti-goat IgG, respectively. The antibody staining was visualized and imaged under a laser scanning confocal microscope (Zeiss LSM510, Oberkochen, Germany).

### Statistical analysis

Data are expressed as mean ± SEM. The differences among groups were assessed by ANOVA. When the *F* statistic was significant, the mean values obtained from each group were then compared by Fisher least significant difference method. A *P value* <0.05 was considered statistically significant. The statistical analysis was performed using SPSS for Windows 13.0.
